# Peak response regularization for localization

**DOI:** 10.1038/s41598-024-65770-2

**Published:** 2024-06-28

**Authors:** Jiawei Yu, Jinzhen Yao, Chuangxin Zhao, Xianhong Zhao, Qintao Hu

**Affiliations:** 1 AVIC Chengdu Aircraft Industrial(Group)Co., Ltd., Chengdu, 610092 China; 2 University of Chinese Academy of Sciences, Beijing, 100039 China

**Keywords:** Electrical and electronic engineering, Computer science, Information technology, Statistics

## Abstract

Deep convolutional neural networks approaches often assume that the feature response has a Gaussian distribution with target-centered peak response, which can be used to guide the target location and classification. Nevertheless, such an assumption is implausible when there is progressive interference from other targets and/or background noise, which produces sub-peaks on the tracking response map and causes model drift. In this paper, we propose a feature response regularization approach for sub-peak response suppression and peak response enforcement and aim to handle progressive interference systematically. Our approach, referred to as Peak Response Regularization (PRR), applies simple-yet-efficient method to aggregate and align discriminative features, which convert local extremal response in discrete feature space to extremal response in continuous space, which enforces the localization and representation capability of convolutional features. Experiments on human pose detection, object detection, object tracking, and image classification demonstrate that PRR improves the performance of image tasks with a negligible computational cost.

## Introduction

**Figure 1:**
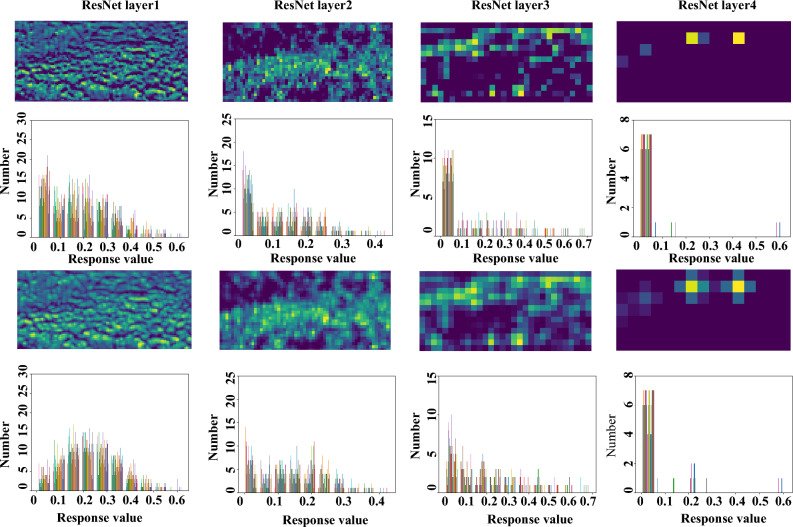
Feature response map (first and third rows) and histograms of feature response without (second row) and with (fourth row) (Best viewed in color and with zoom).

During the past few years, convolutional neural networks (CNNs) have achieved rapid development and breakthroughs in the past decade, including image classification^1–3^, object detection^4,5^, object tracking^6–8^, automatic driving^9,10^. By continuously arranging and combining different CNN layers, pooling and activation functions, CNNs can obtain different levels of features and global semantic information^11,12^. Although CNNs have become the most excellent model in object detection tasks, there are still many problems in actual use. Their performance in precise target positioning is still limited due to the following 2 reasons:

1. To achieve the invariance of target movement and deformation, and reduce the amount of computation, the CNNs use multiple pooling operations to reduce the spatial resolution, extract high-level semantic features, and confuse the spatial location of features, but also lose some information of network features^13–15^;
2. Many factors, including multi-target occlusion, appearance variance and/or background noise, remain challenging state-of-the-art CNN-based methods. As shown in Fig. 1(up), The intermediate features of visual tasks will be seriously lost with the deepening of features, and the target-centered assumption has been challenged^16^.

To conquer the issues, batch normalization (BN) is an effective method to normalize features^16^, which can avoid gradient explosion and gradient disappearance, and also improve the recognition ability of network features. RetinaNet^17^ suggests using focus loss to improve the imbalance between positive and negative samples during training to strengthen feature learning. SENet^18^ used squeezing and stimulation operations on the channels of neural network, which can automatically learn the importance of different channel characteristics. Dilated convolution^19^ is designed to increase the receptive field and improve the positioning accuracy of small targets. Although these methods have achieved good results, inaccurate or even incorrect positioning will still occur for the above problems. The existing methods usually carry out specific post-processing on the feature map, the feature distribution is still not a perfect Gaussian distribution, which will affect the learning efficiency of the network and neglect the regularization of the feature space. There are few studies on direct spatial regularization of convolution features^20–22^.

Therefore, our aim is to enhance the localization and representation of convolution features in a straightforward and efficient manner. We propose employing a simple-yet-effective cross maximization as the peak response regularization method to execute and adjust the extreme response, as well as to suppress the sub-extreme response. It can work as a plug-and-play module in computer vision tasks to adjust the shallow and deep features in the neural network and improve the performance of multiple tasks without increasing the amount of computation. Finally, the effectiveness of our algorithm is verified by human pose detection, object detection, object tracking and image classification tasks.

The remainder of this paper is summarized as follows. Related works are described in “Related work” section and the proposed PRP approaches are respectively presented in "Peak response regularization (PRR)" section. Experimental results are given in “Experiment” section. We conclude this paper in “Conclusion” section.

## Related work

By incorporating the spatial relation of features, CNN has been an effective model for spatial localization tasks including visual object tracking^23–25^, human pose detection^26,27^, object detection^17,28^ and image classification^18,29^. Nevertheless, CNNs developed for these tasks usually focus on finding discriminative representation through extensive offline learning but unfortunately overlook variance of feature response and inference from objects’ context area.

### Image classification

Image classification is a classic task. Dilated convolution is designed to increase the receptive field and improve the positioning accuracy of small targets. SENet^18^ leverages a squeeze-and-excitation operation to filter out local extremal features while enforcing feature representation. Nevertheless, it don’t involve any spatial regularization of convolutional features. Although SENet can enforce the discrimination capability of channels, it has negligible impact on features.

### Human pose detection

In human pose detection, most methods model key points as Gaussian distribution. OpenPose^26^ achieves accurate key-point localization by using Part Affinity Fields. In the following work^27^, a Part Intensity Field (PIF) and a Part Association Field (PAF) are proposed to associate body parts to form full human poses. High-resolution network and hourglass network are also used to achieve precise keypoint localization^30^.

### Object detection

In object detection, point-based methods^31,32^ use corner points to detect objects while leveraging corner/center pooling to align features and improve object localization. FreeAnchor^33^ incorporates a learning-to-match mechanism to break IoU restriction, allowing objects to localize anchors/features in a flexible manner. Dilated convolution^19^ is designed to increase receptive fields while improving the localization precision of small objects.

### Object tracking

Object tracking is formulated in a metric (similarity) learning framework, assuming that the object response has a Gaussian distribution with a target-centered peak response to facilitate state estimation. Classification and state estimation are integrated into a Siamese network^34^ to measure the similarity between the target and the candidates for tracking. Semantic branches and appearance branches are constructed in a dual Siamese network^35^, and saliency mechanisms are introduced in the attention-based Siamese network^36^. SiamRPN^24,37^ combines the Siamese network with region proposal network (RPN), allowing trackers to estimate target extent when positioned accurately. SiamRPN++^24^ introduces a deeper feature network into the SiamRPN^37^, which successfully enables the Siamese network to perform end-to-end offline pre-training on ResNet^29^.

Despite the effectiveness of various object/pose localization approaches, direct spatial regularization of convolutional features is seldom explored. Existing approaches usually use specific post-processing on feature maps but unfortunately ignore feature spatial regularization. In this paper, we propose Peak Response Regularization (PRR) and aim to enforce both the localization and representation capability of convolutional features in an efficient manner.

## Peak response regularization (PRR)

**Figure 2:**
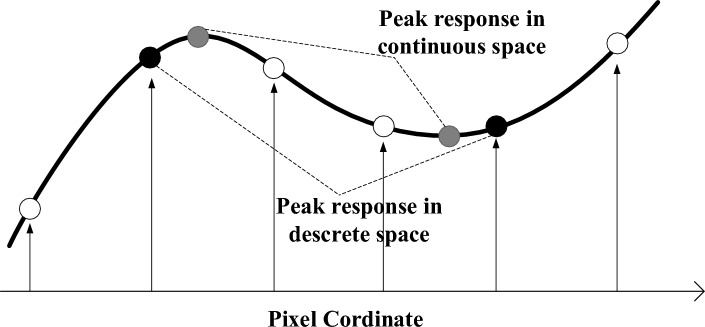
Illustration of continuous and discrete extremal values (peak response).

**Figure 3:**
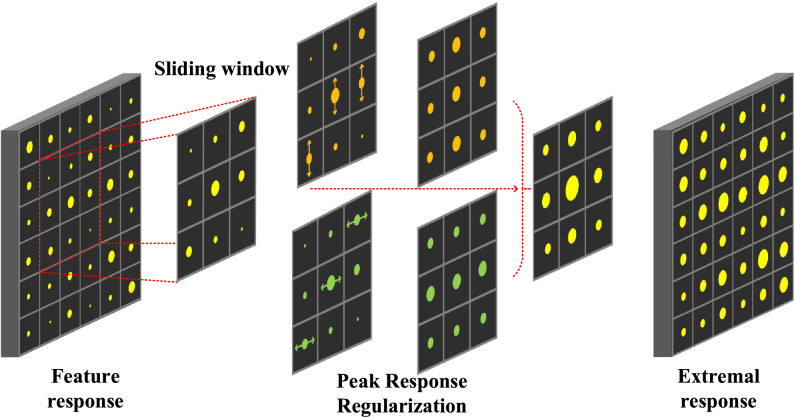
Illustration of the proposed G-SPS approach, cross maximization to enforce and align extremal response, as well as suppressing sub-extremal response with slide window. (Best viewed in color).

Feature extraction, coding and fusion of visual features are important components of semantic image analysis methods. The development of convolutional neural network combines these traditional independent steps. The effectiveness of visual tasks depends on the effectiveness of these steps. Object detection is to use convolution neural network for feature extraction and encode the target into Gaussian feature model. Accurate object localization is crucial in many computer vision tasks including human pose detection, visual object detection, object tracking, and image recognition. Despite the unprecedented performance achieved by CNNs over object localization, they remain challenged by the variance of object appearance and inference from backgrounds.

### Peak response in continuous space

The peaks on a feature response map are in discrete space instead of continuous space. It requires converting the peaks from discrete space instead of continuous space to identify the most stable features, as shown in Fig. 2. To fulfill this purpose, we first model the peak response from a perspective of extremal point detection in continuous space. Let $z=(p,q)$ and *f*(*z*) denote a pixel location and a response value on the feature response map, respectively. Based on Taylor expansion, we have that

1 $$\begin{aligned} f(z)=f+\frac{\partial f^{T}}{\partial z}z+\tfrac{1}{2}z^{T}\frac{\partial ^2 f}{\partial z^2}z. \end{aligned}$$

To get the extreme value of *f*(*z*) in terms of location variable *z*, it requires to set the derivative on *z* to zero, as

2 $$\begin{aligned} \frac{\partial f(z)}{\partial z}\approx \frac{\partial f}{\partial z}+\frac{\partial ^2 f}{\partial z^2}\widehat{z}=0, \end{aligned}$$

and we have

3 $$\begin{aligned} \widehat{z}=-\frac{\partial ^2 f^{-1}}{\partial z^2}\frac{\partial f}{\partial z}, \end{aligned}$$

and the extremal response value is calculated as

4 $$\begin{aligned} f(\widehat{z})=f+\tfrac{1}{2}\frac{\partial f^{T}}{\partial z}\widehat{z}. \end{aligned}$$

### Response regularization

To identify peak response in continuous space, we approximate $\frac{\partial f^{T}}{\partial z}\widehat{z}$ by respectively maximizing response values in horizontals and vertical (row and column) orientations, Fig. 3. On the feature map predicted, PRP is first performed to concentrate the feature map into a horizontal pooling map. This procedure is done by finding the maximum feature in each row of the feature map and assigning all pixels in the line the maximum feature value. In a similar way, vertical PRP is performed in each column on the feature map to obtain the vertical pooling map. The horizontal and vertical pooling maps are summarized, as

5 $$ \begin{aligned}   \hat{f}_{{pq}}  =  & \max \left( {f_{{p(q - i//2)}} ,f_{{p(q - i//2 + 1)}} , \ldots ,f_{{pp(q + i//2)}} } \right) \\     & \quad  + \max \left( {f_{{(p - i//2)q}} ,f_{{(p - i//2 + 1)q}} , \ldots ,f_{{(q + i//2)q}} } \right), \\  \end{aligned}  $$

where $f_{pq}$ denotes the response value at the location (*p*,*q*) and *i* the size of sliding window. According to Eq. 4, the feature values are converted to

6 $$\begin{aligned} f(\widehat{z})=\alpha \left(f+\tfrac{1}{2}\widehat{f}_{pq}\right), \end{aligned}$$

where $\alpha$ is a normalization factor, which is experimentally set to be 0.5.

### Object localization

**Figure 4:**
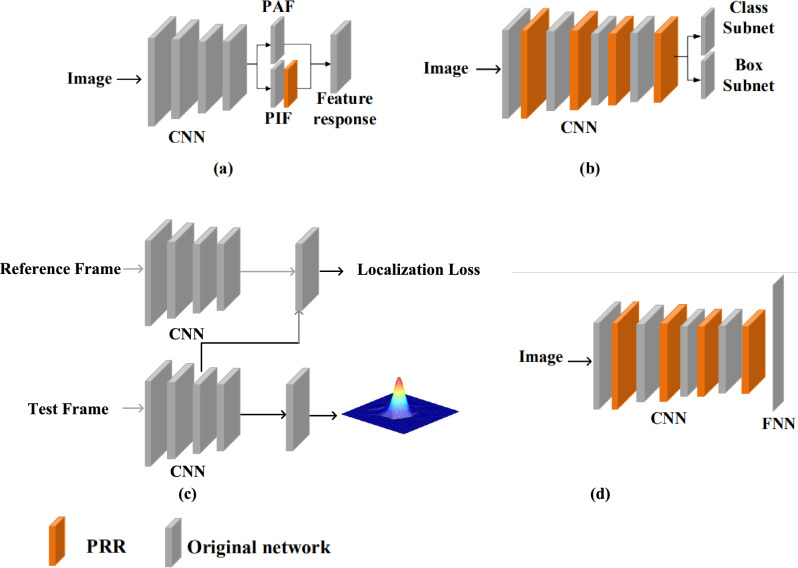
Network architecture with Peak Response Regularization (PRR) for human pose detection, object detection and image classification.

The proposed PRR is applied on feature response maps for typical object detection tasks including human pose detection, visual object detection and image classification, Fig. 4. For different tasks, different sliding window sizes are adopted for PRR. For object tracking, we define the window size as large as the size of feature map, which can suppress the sub-peak of response map and reduce the drift of the model. In human pose detection, the size of sliding window is set to be small (5$\times$5) to capture different local maxima (representing joints of different human bodies). In object detection, the size of sliding windows is very small (3$\times$3). Considering that in the single-stage object detection framework, each single deep pixel is used to represent an object, a 3$\times$3 rectangle region can cover both the object and its context area.

### Human pose detection

Human pose detection defines a keypoint coding problem by using the Gaussian mixture distribution prior. The key to human pose detection lies in precise keypoint localization. Unlike object tracking, there are multiple local maxima on the keypoint response map. When performing pose detection, sub-peak response around keypoints could produce interference and reduce the precision of keypoint localization. To enforce keypoint detection, PRR is applied on the response map to regularize feature distribution, Fig. 4a. The response map is produced by a state-of-the-art human pose detection approach^27^, which uses a Part Intensity Field (PIF) to localize keypints and a Part Association Field (PAF) to estimate human pose on detected keypoints.

### Object detection

CNN-based object detection typically consists of an object classification module and an object localization module. While the classification module aims to classify a region of convolutional feature maps into object categories, the localization module predicts object extent via a bounding-box regression procedure^17^. During the procedure, object appearance and background noise lead to variance of peak response, which deteriorates object classification and/or localization. PRR is therefore applied after each convolutional layer to regularize peak response while enforcing feature representation, Fig. 4b.

### Object tracking

Object tracking usually assumes that the feature response has a Gaussian distribution with target-centered peak response^25,34^. Nevertheless, such an assumption is implausible when there is progressive interference from other targets and/or background noise, which produces sub-peaks on the tracking response map and causes model drift. To mitigate the interference, PRR is applied to aggregate and align discriminative features to modify the tracking response to Gaussian distribution, as shown in Fig. 4c.

### Image classification

Image Classification is a fundamental problem in computer vision, which aims to classify images based on pre-trained network models^18,29^. Although image classification does not explicitly involve object localization, PRR on feature response maps could also benefit feature representation and improve classification performance. Like object detection, PRR is applied after each convolutional layer to align most discriminative feature representation to local peaks, Fig. 4d.

## Experiment

### Implementation details

In this section, we evaluate the performance of object tracking, human pose estimation, object detection and classification network with and without using PRR. Experiments are carried out with Pytorch on Intel Xeon E5-2678 V3 CPU with 2.5GHz*48 and Nvidia GTX 2080ti GPU$\times$4 with 11GB$\times$4 memory.

### Peak response regularization

**Figure 5:**
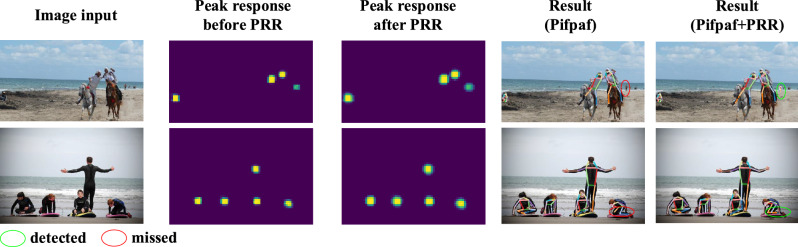
Human pose detection with/without PRR on the MS-COCO 2017 dataset. With PRR, the response map is smoothed and can better fit the Gaussian distribution priors (supervision), which facilities keypoint detection.

PRR targets regularizing the peak response from discrete space to continuous space. In Fig. 5, the peak response with/without PRR is compared. It can be seen that with PRR, the response map is smoothed and can better fit the Gaussian distribution priors (supervision), which facilitates keypoint detection. In the deep learning framework, better fitting of supervision usually means easier training of the network model and higher performance. In Fig. 1, the response feature maps for object detection are compared.

It can be seen that after PRR, the response feature maps are regularized to form distributions centered at local response peaks, particularly in deeper fourth convolutional layers. Such feature distribution could be more robust to object appearance variance and noise inference. By comparing feature histograms before and after PRR, we can see that PRR can reduce the low response features while enforcing local peak response. After PRR, the global histogram is regularized to enforce the effective (larger) features while depressing trivial ones.

**Table 1:** Comparison of pose detection performance and time cost on the MS-COCO 2017 dataset.

Backbone	Detector	*AP*	$t_{total}[ms]$	$t_{dec}[ms]$
ResNet-50	PifPaf^27^	62.6	**79**	39
ResNet-50	PifPaf+PRR (ours)	**64.3**	80	39

Significant values are in bold.

**Table 2:** Comparison of object detection performance and and time cost on the MS-COCO 2017 dataset.

Backbone	Detector	*AP*	$AP_{50}$	$AP_{75}$	$AP_{S}$	$AP_{M}$	$AP_{L}$	*Time*[*ms*]
ResNet-50	RetinaNet^17^	35.7	55.0	38.5	18.9	38.9	46.3	**153**
ResNet-50	RetinaNet+PRR	**37.3**	**56.4**	**39.9**	**21.4**	**40.9**	**49.6**	154

Significant values are in bold.

### Performance and comparison

#### Precision

On human pose detection, we test the proposed PRR approach on the MS-COCO 2017 dataset by adding PRR after the PIF module of the PifPaf^27^. In Fig. 5, it can be seen that with PRR local response peaks are regularized to be more continuous. The regularized peak response can fit Gaussian distribution prior (supervision) better and get more accurate keypoint localization. As a result, our approach improves the average precision (AP) value by 1.7% (62.6–64.3%), Table 1, which is a significant margin for the challenging pose detection task. As shown in the first row of Fig. 5, the baseline PifPaf approach misses a small-scale person. In contrast, our approach detects the small person for the improved localization capability of regularized feature response.

**Figure 6:**
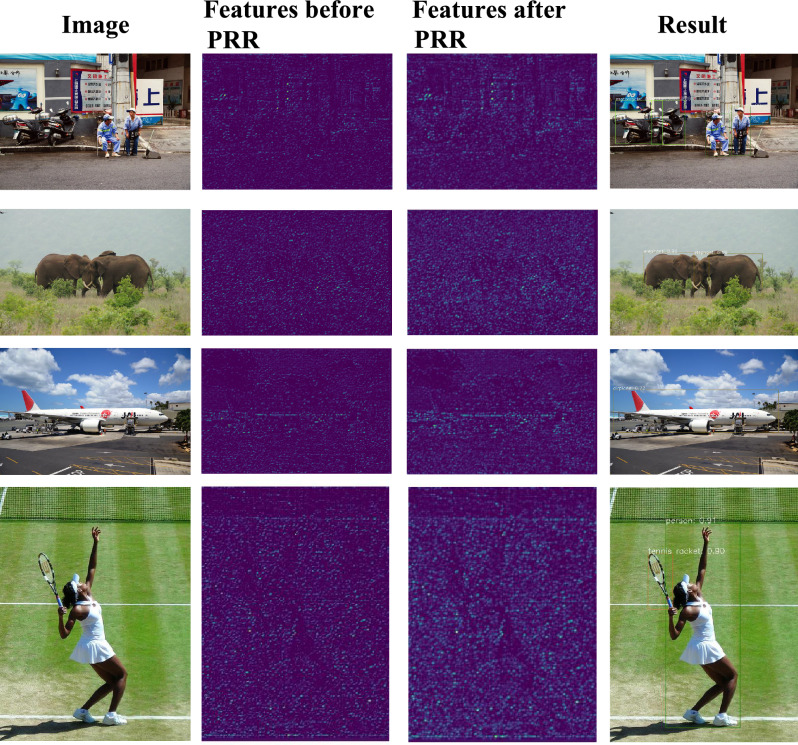
Object detection with/without PRR on the MS-COCO 2017 dataset. With PRR, the response map is more salient to extract more features to assist location.

For object detection, RetinaNet^17^ with ResNet-50 is selected as the baseline and the proposed PRR is added into each convolutional layer of the backbone ResNet. As shown in Table 2 and Fig 6, on the MS-COCO 2017 dataset the introduction of PRR improves the AP value by 1.6% (35.7–37.3%), which again validates the effectiveness of the proposed approach.

For object tracking, ATOM tracker^25^ with a target classification branch and a target localization branch. The classification branch converts the feature map into a response map and provides the coarse locations of the target. Upon the classification branch, the PRR is applied in a plug-and-play manner to regularize the response map to Gaussian distribution. On the VOT2018^38^ dataset, can improve EAO by 0.19 (0.420 vs. 0.401), as reported in Table  3.

**Table 3:** Tracking performance comparison on VOT-2018.

Tracker	EAO	Accuracy	Robustness	FPS
ATOM+PRR	0.420	0.609	0.191	39
ATOM^25^	0.401	0.590	0.204	40

For classification, by adding PRR to ResNet50, we improve the image classification accuracy by 0.9% (76.2–77.1%) on the ImageNet^39^ dataset.

#### Time cost

As reported in Table 1, we can see that our proposed method only takes a slightly longer time than PifPaf to detect human pose, specifically, PifPaf takes 79 ms on average and our approach takes 80 ms. In Table 3, with a single GPU, the proposed PRR achieves a tracking speed of 39 fps. Compared with the speed (40 fps) of the baseline ATOM. In Tables 2 and 4, we further validate that PRR achieves significant detection and classification performance gains with negligible computational cost.

**Table 4:** Comparison of image classification performance and time cost on the ImageNet dataset.

Backbone	Top-1 Acc.	Top-5 Acc.	*Time*[*ms*]
ResNet-50^29^	76.2	92.9	**5**
ResNet-50+PRR	**77.1**	93.4	**5**

Significant values are in bold.

## Conclusion

Precise object localization is a primary problem in many computer vision tasks including object tracking, object detection, and human pose detection. Nevertheless, the localization of the target object remains challenged by interference from nearby objects, object appearance variation, and background noise. In this paper, we propose a peak response modeling approach and alleviate the localization inference from the perspective of feature response regularization. A plug-and-play Peak Response Regularization (PRR) is proposed to convert local extremal response in discrete feature space to continuous space to aggregate and align discriminative features. The proposed feature response regularization improves the performance of object tracking, image classification, pose and object detection, with shirking contrast with the baseline approaches. The proposed approach can provide a new insight for object localization with convolutional features.

## Data Availability

Deidentified data analyzed in this study will be available to share upon request to the corresponding author.
